# Serial Cardiac Magnetic Resonance Imaging in Patients with Mitral Valve Prolapse—A Single-Center Retrospective Registry

**DOI:** 10.3390/jcm13092669

**Published:** 2024-05-02

**Authors:** Maarten Blondeel, Wouter L’Hoyes, Tomas Robyns, Peter Verbrugghe, Pieter De Meester, Tom Dresselaers, Pier Giorgio Masci, Rik Willems, Jan Bogaert, Bert Vandenberk

**Affiliations:** 1 Department of Cardiology, University Hospitals Leuven, 3000 Leuven, Belgium; 2 Department of Cardiovascular Sciences, KU Leuven, 3000 Leuven, Belgium; 3 Department of Cardiac Surgery, University Hospitals Leuven, 3000 Leuven, Belgium; 4 Department of Radiology, University Hospitals Leuven, 3000 Leuven, Belgium; 5 Department of Imaging and Pathology, KU Leuven, 3000 Leuven, Belgium; 6 School of Biomedical Engineering and Imaging Sciences, King’s College London, St Thomas Hospital, London SE1 7EH, UK

**Keywords:** mitral valve prolapse, mitral annular disjunction, cardiac magnetic resonance imaging, sudden cardiac death, ventricular arrhythmias, implantable cardioverter-defibrillator, mitral valve surgery

## Abstract

**Background:** Mitral valve prolapse (MVP) and mitral annular disjunction (MAD) are common valvular abnormalities that have been associated with ventricular arrhythmias (VA). Cardiac magnetic resonance imaging (CMR) has a key role in risk stratification of VA, including assessment of late gadolinium enhancement (LGE). **Methods:** Single-center retrospective analysis of patients with MVP or MAD who had >1 CMR and >1 24 h Holter registration available. Data are presented in detail, including evolution of VA and presence of LGE over time. **Results:** A total of twelve patients had repeated CMR and Holter registrations available, of which in four (33%) patients, it was conducted before and after minimal invasive mitral valve repair (MVR). After a median of 4.7 years, four out of eight (50%) patients without surgical intervention had new areas of LGE. New LGE was observed in the papillary muscles and the mid to basal inferolateral wall. In four patients, presenting with syncope or high-risk non-sustained ventricular tachycardia (VT), programmed ventricular stimulation was performed and in two (50%), sustained monomorphic VT was easily inducible. In two patients who underwent MVR, new LGE was observed in the basal inferolateral wall of which one presented with an increased burden of VA. **Conclusions:** In patients with MVP and MAD, repeat CMR may show new LGE in a small subset of patients, even shortly after MVR. A subgroup of patients who presented with an increase in VA burden showed new LGE upon repeat CMR. VA in patients with MVP and MAD are part of a heterogeneous spectrum that requires further investigation to establish risk stratification strategies.

## 1. Introduction

Mitral valve prolapse (MVP) is the most frequent valvular abnormality with an estimated prevalence up to 3% in the general population, and often coincides with mitral annular disjunction (MAD) [1]. A small subset of patients with MVP present with severe ventricular arrhythmias and sudden cardiac death (SCD). While the incidence for severe arrhythmic events in patients with uncomplicated MVP is rather low at 0.3%/year (95% CI 0.1–0.8), higher incidences up to 4%/year (95% CI 2–9) have been reported in patients with arrhythmic mitral valve prolapse (AMVP) [2]. These reports resulted in an increased attention for risk stratification for SCD in this population [3,4].

In 2022, the European Heart Rhythm Association (EHRA) released an expert consensus statement proposing a multimodality approach for risk stratification of SCD in patients with arrhythmic MVP (AMVP) and MAD [3]. The document proposes useful guidance for risk stratification, including electrocardiographic monitoring and multimodal cardiovascular imaging with echocardiography and cardiac magnetic resonance imaging (CMR). However, several evidence gaps remain including how often risk stratification should be reassessed as well as the potential role for cardiac surgery [3].

The aim of this retrospective analysis was to report the clinical evolution of the small subset of patients with MVP or MAD included in our registry who underwent serial CMR [4].

## 2. Materials and Methods

### 2.1. Study Population

This single-center retrospective analysis includes all patients in our MVP and MAD registry who underwent >1 CMR with assessment of late gadolinium enhancement (LGE) and >1 24 h Holter recording at our institution between January 2008 and September 2023. The design and outcome of our single-center MVP registry have been published previously [4]. In brief, all consecutive patients with MVP and/or MAD who underwent a CMR at our institution were included in the registry. Exclusion criteria were (1) prior surgical mitral valve intervention; (2) presence of an alternative well-defined arrhythmic substrate (coronary artery disease, underlying cardiomyopathy, primary arrhythmic disease, complex congenital heart disease…); and (3) insufficient clinical data available to complete the risk assessment. This study was performed in accordance with the Declaration of Helsinki. The study was approved by the UZ Leuven ethical committee and given the retrospective study design the need for informed consents was waived.

### 2.2. Baseline and Follow-Up Clinical Data

Clinical data, including demographics, pharmacological changes and electrocardiographic evolution, were collected by reviewing the electronic medical record as reported previously [4]. In brief, the clinical presentation, medical history, and medication were collected at the time of baseline CMR. Follow-up data were collected by reviewing all available reports, including electrocardiographic data, and reports from electrophysiological and surgical interventions. Ventricular arrhythmias were assessed by reviewing all ECGs, ambulatory heart rhythm monitoring, cardiopulmonary exercise testing, and cardiac implantable electronic device interrogations, including implantable cardioverter defibrillator (ICD) therapies. Exams were reviewed for the presence of premature ventricular complex (PVC), non-sustained VT (nsVT), and sustained ventricular tachycardia (VT). In this study, only 24 h Holter recordings from which a detailed arrhythmia burden could be calculated were included. However, also long-term ambulatory recordings were performed and while these may have impacted clinical decision-making, these could not be included. Ambulatory heart rhythm monitoring recordings were analyzed using Synescope (Microport, Shanghai, China). Results of invasive diagnostic electrophysiological (EP) studies with programmed ventricular stimulation (PVS) were reviewed. Sustained VT was defined as VT lasting ≥30 s or requiring immediate termination because of hemodynamic compromise. nsVT was defined as ≥3 consecutive ventricular beats at a rate of ≥100 beats per minute lasting up to 30 s and classified as high-risk if polymorphic or if the rate ≥180 beats per minute [3,5] AMVP was defined as a total PVC burden ≥5%, nsVT, VT, or ventricular fibrillation [3].

### 2.3. CMR Imaging Protocol and Analysis

CMR studies were performed on 1.5 T whole-body scanners with electrocardiographic triggering and a cardiac-dedicated phase array coil (Achieva (2008–2014) and Ingenia (2015–2019); Philips Healthcare, Best, The Netherlands) and analyzed using a dedicated workstation (IntelliSpace, Philips Medical Systems, Best, The Netherlands and SuiteHEART, Neosoft, Pewakee, WI, USA). All studies fulfilled the EHRA criteria for a comprehensive CMR protocol and were analyzed by an EACVI-CMR level III accredited reader [3]. Studies included cine imaging for anatomic and functional assessment of the left and right ventricles in a short-axis stack, and standard 2-, 3-, and 4-chamber views using a balanced steady-state free-precession sequence. Typical imaging parameters were repetition time/echo time 3.6/1.8 ms; sense factor 2; flip angle 60°; section thickness 8 mm; matrix 256 × 164; field of view 350 mm; pixel size 1.3 × 1.6 mm; number of phases 30 and phase percentage 67%. The ventricular endocardial and epicardial borders were manually contoured at end-systole and end-diastole using the short-axis cine stack to quantify ventricular dimensions and function. The visualizations of the MV were optimized using a stack of 5 consecutive slices in a 3-chamber view. Individual mitral valve scallops were assessed in cine-CMR images acquired in the standard 2-, and 4-chamber, as well as a stack of 3-chamber views perpendicular to the short-axis of the mitral annular major axis, centered at the aortic outflow track. MVP was assessed in 3-chamber view and defined as a superior displacement ≥2 mm beyond the mitral annulus plane of any part of the mitral leaflet [6]. MAD was defined as an end-systolic separation in longitudinal view of the mitral annulus and ventricular myocardium [3,7]. MAD was quantified as the longitudinal length in the long axis view [3,7]. The severity of MVP was corrected for the length of the MAD, if MAD was present [4]. Phase-contrast velocity-encoded CMR imaging of the aortic outflow tract was performed to quantify aortic forward flow and indirectly quantify MR volume. The regurgitant fraction of mitral valve regurgitation was calculated by dividing the regurgitant volume (difference between left ventricular stroke volume and forward aortic flow) by the left ventricular stroke volume. The regurgitant fraction was graded as: 0 (0–5%); 1 (5–16%); 2 (16–25%); 3 (26–48%); and 4 (>48%) [8]. An enlarged left atrium was defined as a left atrial area >15 cm^2^/m^2^ [9].

All studies included assessment of late gadolinium enhancement (LGE). LGE images were acquired 10 min after intravenous gadolinium contrast administration (gadoterate meglumine 0.15 mmol/kg (2008–2015) or gadobutrol 0.075 mmol/kg (2016–2023)) and obtained in SA, 2-, 3- and 4-chamber orientations, entirely encompassing both ventricles, with no slice gap. LGE imaging included breath-hold 2D and 3D turbo field-echo inversion recovery and (dark-blood) phase-sensitive inversion-recovery sequences. The optimal inversion time was determined using a Look-Locker sequence. The presence of LGE was scored visually and classified as subendocardial, midwall, subepicardial, or transmural. Subsequently, LGE was quantified as the ratio between LGE mass and left ventricular mass, hence expressed in %. LGE location was assessed using the 17-segment heart model by the American Heart Association [10].

### 2.4. Statistical Analysis

Statistical analysis was performed using SPSS (IBM Statistics, version 28, IBM Corp. Armonk, NY, USA). Continuous variables were presented as a median with quartiles 1 and 3, or range (minimum–maximum) whichever is specified. Categorical variables were presented as frequency and proportion. Baseline comparisons of patients who underwent >1 CMR and those who underwent 1 CMR were performed using Mann–Whitney U test for continuous variables and 2-sided Fisher’s Exact test for categorical variables. A *p*-value < 0.05 was considered significant. Given the low sample size, the remainder of the analysis was considered merely descriptive. An intra- and interobserver variability assessment for LGE measurements was performed using Bland–Altman plots (GraphPad Prism 9.5.1, GraphPad Software LLC, La Jolla, CA, USA) reporting bias and the 95% limits of agreement in Supplementary Figure S1.

## 3. Results

### 3.1. Study Population

Of 169 patients in the retrospective registry, 12 (7.1%) had >1 CMR and >1 24 h Holter recording available during follow-up. A comparison of patients referred for repeat CMR with those who underwent only one CMR is presented in Supplementary Table S1. The median age was 44 years (range 20–60) and nine (75.0%) were female. Patients referred for repeat CMR were non-significantly younger at the first CMR, had a longer overall follow-up, had more frequently a history of heart failure, higher use of beta-blocking drugs, and had more often a PVC on any ECG. The indications for the baseline CMR were ventricular arrhythmias (n = 7, 58.3%), left ventricular (LV) remodeling assessment (n = 4, 33.3%) of which one patient had Marfan disease. Barlow’s disease was present in five patients (41.2%). While one patient (8.3%) never fulfilled the criteria for AMVP, ten patients (83.4%) fulfilled the AMVP criteria before the baseline CMR and one patient (8.3%) only fulfilled AMVP criteria at the second CMR. Four (33.3%) patients underwent surgical mitral valve repair using a minimal invasive approach, all for moderate-to-severe mitral valve regurgitation.

### 3.2. Serial MRI in Patients without Surgical Intervention

A total of eight (66.7%) patients underwent a repeat CMR after a median of 4.7 years (range 2.7–7.7). Summarized details are presented in Table 1. The median age was 52 years (range 20–60) and six were female (75.0%). MVP was present in seven (87.5%), predominantly bileaflet MVP (62.5%). All patients had MAD with a median length of 7 mm (range 6–10). At baseline, three (37.5%) patients had LGE, either myocardial (segment 5) or in the posterior papillary muscle.

At baseline, a 24 h Holter was obtained a median of 0.3 months before CMR. Of these, five out of eight (62.5%) were performed prior to the CMR, while two (25.0%) were performed on the same day as the CMR in the work-up of the arrhythmia. Only one 24 h Holter was performed 0.8 months after the CMR where the findings on CMR triggered an ambulatory rhythm monitoring.

**Figure 1:**
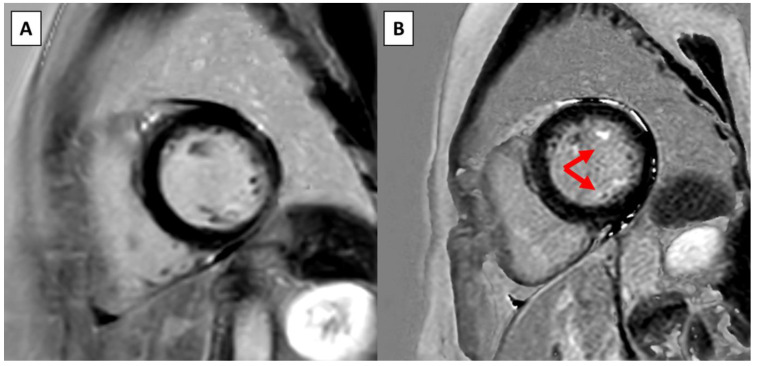
Example of new LGE on serial CMR in a patient without surgical intervention. (**A**) Short-axis view on the baseline CMR at the level of the papillary muscles. (**B**) Short-axis view at the same level approximately 4 years later shows new LGE in both papillary muscles (red arrows).

At follow-up CMR, six out of eight (75.0%) 24 h Holter recordings were performed prior to the follow-up CMR resulting in a median time of 2.2 months before CMR. In three (37.5%) cases the Holter triggered the repeat CMR, while in the remaining cases the Holter was obtained in the routine follow-up. Upon follow-up CMR, four (50.0%) patients had a new LGE present. New myocardial LGE was present in the basal inferolateral segment of one patient. New papillary muscle LGE was present in three patients, and all of them had new LGE in both papillary muscles (Figure 1) and one of these had new LGE in both the basal and mid inferolateral segments and both papillary muscles (Supplementary Figure S2). The change in LGE from baseline is presented in Supplementary Figure S3.

From a clinical perspective, one patient remained free from arrhythmias and one patient underwent catheter ablation for symptomatic PVC ectopy from the posterior papillary muscle despite treatment with beta-blockers and flecainide. The catheter ablation reduced the PVC burden from 5% to 2%. Four patients (50.0%) presented with either increased arrhythmia burden, presyncope or syncope. In one patient without any LGE who presented with syncope, the PVS was negative. One patient with baseline LGE in the posterior papillary muscle and new LGE in the basal inferolateral segment presented with an increase in PVC and nsVT burden and also had a negative PVS. In both these patients, the ventricular arrhythmias were appropriately suppressed with beta-blockers. Two patients had positive PVS and received an ICD. The first patient presented with nsVT and presyncope during exercise testing and had a new LGE in both papillary muscles. The second patient presented with presyncope and had a new LGE in both the basal and mid inferolateral segments and in both papillary muscles. None of them have received any ICD therapy under treatment with either betablockers or betablockers and flecainide, respectively. Upon repeat CMR, one patient showed disappearance of mitral valve regurgitation. At baseline, this patient had a mildly reduced LVEF with grade 1 mitral regurgitation, no LGE, and fulfilled the AMVP criteria before baseline CMR based on documented nsVT on Holter. Upon repeat CMR she was treated with betablockers and low dose ACE-inhibitors, and no mitral valve regurgitation was noted. The LVEF evolved from 48% to 53% upon repeat CMR.

### 3.3. Serial MRI in Patients with Surgical Intervention

A total of four (33.3%) patients underwent a repeat CMR after a median of 10 months (range 3–48) after minimal invasive mitral valve repair. Summarized details are presented in Table 2. All baseline CMRs were performed for evaluation of arrhythmias. The median age was 39 years (range 36–55) and three were female (75.0%). All patients had MVP, predominantly involving both leaflets (75.0%), and two (50.0%) had MAD. With surgery, MAD was corrected in both cases.

At baseline CMR only one patient had LGE present in the mid inferolateral segment. At baseline, a 24 h Holter was obtained a median of 1.3 months before CMR, and in only one case the Holter was performed 1 week after the CMR. Half of the repeat CMRs were performed due to arrhythmias, and this corresponds to the indication of the repeat Holter. The repeat Holter was obtained a median of 0.7 months after the repeat CMR.

**Figure 2:**
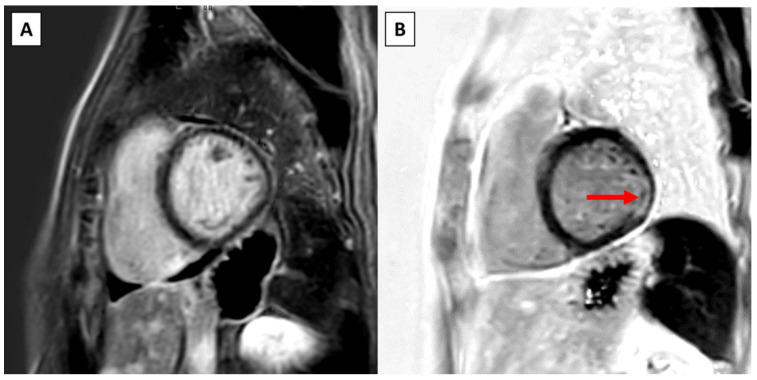
Example of new LGE on serial CMR in a patient with surgical intervention. (**A**) Short-axis view on baseline CMR. (**B**) Short-axis view at the same level within 1 year shows new LGE in the basal inferolateral left ventricular wall (red arrow) and diffuse pericardial enhancement.

Upon follow-up CMR, two (50.0%) patients had a new LGE present, both in the basal inferolateral segment (Figure 2 and Supplementary Figure S4). There was no new LGE in the papillary muscles. The change in LGE from baseline is presented in Supplementary Figure S3.

From a clinical perspective, one patient did not have ventricular arrhythmias after mitral valve repair. In one patient with a new LGE, the PVC burden decreased from 3.8 to 1.8% under therapy with beta-blockers. Two patients had an increase in PVC burden and nsVT. The first patient, who had unchanged LGE in the mid inferolateral segment, presented with an ischemic stroke 2 months after uneventful mitral valve repair. Rhythm monitoring did not show any atrial fibrillation, but intermittently a very high burden of PVCs up to 24.3% and 569 runs of nsVT. The PVC burden decreased during follow-up and the unchanged dose of beta-blockers. The second patient, who had a new LGE in the basal inferolateral segment, underwent a repeat CMR 3 months after surgery for an increase in PVC burden, from 4.9% to 6.6%, which was noticed during a cardiac rehabilitation program. Since this patient was asymptomatic, no intervention occurred, and the PVC burden decreased during follow-up while on unchanged dose of beta-blockers.

## 4. Discussion

MVP and MAD are common valvular abnormalities that may present as a heterogenous spectrum ranging from asymptomatic and benign over symptomatic PVCs and nsVT to life-threatening ventricular arrhythmias and SCD. In this report this heterogenous spectrum is presented with longitudinal follow-up, including serial CMRs. CMR plays a central role in the risk stratification of patients with MVP as it is the study of choice to assess LV remodeling and the presence of fibrosis [3,11,12]. Both LV remodeling and assessment of fibrosis were the main indications for repeating CMR. Patients with repeat CMR were younger at baseline CMR, had a longer overall follow-up since the first cardiac contact, and had more frequently a history of heart failure. While we may not neglect the referral and selection bias when reporting this retrospective analysis, to the best of our knowledge this report is the first to describe serial CMR findings together with arrhythmic outcome in patients with MVP and/or MAD.

In summary, after a median of 4.7 years four out of eight patients with MVP who underwent repeat CMR had new areas of fibrosis. Typically, new fibrosis was located in the papillary muscles and the mid to basal inferolateral wall as has been described before. The vast majority of ambulatory rhythm recordings were performed prior to the CMR; the repeat CMRs were either triggered by documented arrhythmias or in the work-up of LV remodeling. An EP study with PVS was used to guide further management in three of these patients, all of which presented with either syncope or an increase in ventricular arrhythmia burden. Sustained VT could easily be induced in two patients, and both received an ICD. The remaining patient with new fibrosis on CMR was known to have nsVT but is currently treated with amiodarone for atrial fibrillation rhythm control. In one patient with AMVP and mildly reduced LVEF, the repeat CMR showed disappearance of the grade 1 mitral regurgitation at baseline under therapy with beta-blockers and low-dose ACE-inhibitors. When reviewing patients who underwent mitral valve repair, there were two cases with new fibrosis after a relatively short follow-up period. In both cases, new fibrosis was found in the basal inferolateral LV wall. Further, in one case the patient who had fibrosis already present before mitral valve repair, presented with an increase in PVC burden shortly after surgery which settled down later during the follow-up. All surgical procedures were minimal invasive mitral valve repairs limited to intervention of the valve and annulus.

This retrospective analysis illustrates several knowledge gaps in management and risk stratification of ventricular arrhythmias in patients with MVP. First, while the 2022 EHRA expert consensus statement advises to perform periodic clinical evaluation, rhythm monitoring, and echocardiographic assessment, the optimal time window for periodic assessments is unknown [3]. It is common sense that syncope, presyncope, and palpitations may trigger a new assessment. There are no data, however, to support a predefined time frame for serial investigations to timely diagnose asymptomatic ventricular arrhythmias and fibrosis. Second, the value of PVS is unclear. The inducibility of sustained monomorphic VT, as in the two cases presented, is likely more specific. However, the clinical value of a negative test or inducing polymorphic VT or ventricular fibrillation remains unknown. In a retrospective study, Gupte et al. found that one out of five patients with documented symptomatic ventricular arrhythmias, LGE on CMR and negative electrophysiological study had an adverse arrhythmic outcome [13]. Similarly, a systematic review of 22 patients with MVP and documented SCD reported non-inducibility of ventricular arrhythmias in 55% of cases and nsVT in 23% of cases [14]. Third, the evidence on the role of mitral valve surgery to reduce the burden of malignant ventricular arrhythmias is limited and inconsistent as most of the data originate from case series and reports [3]. Also a new onset ventricular arrhythmias after valve surgery have been described. For example, Eckart et al. described a bimodal incidence of sustained monomorphic VT after aortic and mitral valve surgery, occurring either early after surgery or years later [15]. Remarkable in our retrospective analysis was the appearance of a new LGE on the repeated CMR. However, it cannot be ascertained whether the LGE was directly related to the surgical intervention nor to the ventricular arrhythmias. Two recent prospective studies assessed LV remodeling after mitral valve surgery for primary mitral regurgitation using CMR at the 6-months follow-up [16,17]. Craven et al. compared LV remodeling between mitral valve replacement (n = 30) and mitral valve repair (n = 22), but unfortunately the LGE sequences were incomplete or of insufficient quality for direct comparison [17]. Liu et al. studied 105 patients who underwent mitral valve repair and found no change in LGE before and 8.8 months after mitral valve surgery [16]. The studies by Craven et al. and Liu et al. did not report data on arrhythmia, which illustrates the unique findings of our report.

MVP and MAD, particularly patients with AMVP, warrant close follow-up and serial assessment. Risk stratification of ventricular arrhythmias and SCD remains the holy grail of electrophysiology and further efforts to establish an optimal risk stratification strategy should be made. The optimal risk stratification strategy will likely include non-invasive methods to assess the presence of myocardial fibrosis. Recently, Tison et al. successfully developed a deep convolutional neural network to detect LGE on routine 12-lead resting ECGs [18]. Further, Miller et al. correlated areas of myocardial scar with myocardial inflammation using hybrid positron emission tomography and CMR to correlate [19]. This raises the question whether ECG and biomarkers can be used to track myocardial inflammation and predict the presence of myocardial fibrosis. Therefore, prospective multimodal imaging studies with serial assessments are urgently needed to advance the field of risk stratification in patients with MVP.

## 5. Limitations

This retrospective analysis of selected patients from a single-center registry is prone to selection bias. The patients who underwent serial CMR examinations are only a small subset of all patients included in our registry and therefore this analysis lacks statistical power. Further, due to referral and selection bias, the included patients are more likely to present with abnormal results. Therefore, this report should be interpreted taking into account the inherent limitations of retrospective analyses, including the lack of a direct causal relationship between mitral valve surgery, detection of new LGE on CMR, and VA. Due to privacy restrictions, no potentially identifiable information, i.e., patient-level data, could be included. While detailed analysis of the morphology of the ventricular arrhythmias would be of interest, this requires 12-lead ECG documentation of the arrhythmia which was largely unavailable since ventricular arrhythmia burden was assessed using 2-lead Holters.

## 6. Conclusions

Ventricular arrhythmias in patients with MVP and MAD are part of a heterogeneous spectrum. This retrospective analysis illustrates the diverse clinical evolution of MVP and MAD. Repeat CMR was performed in a small group of patients and new areas of fibrosis were found in a subset of these, including shortly after surgical mitral valve repair. A subset of these patients showed an increase in VA. While future prospective research is needed to investigate associations between change in LGE and VA, we believe that for now there should be a low threshold to repeat a CMR in patients with MVP and MAD who present with an increase in ventricular arrhythmias or suspected arrhythmic syncope.

## Figures and Tables

**Table 1:** Changes in CMR and clinical evolution in patients without mitral valve surgery.

Variable	Baseline CMR	Follow-Up CMR
Age (y)	51.3 (19.9–59.5)	55.0 (24.9–67.0)
Female	6 (75.0%)	
History of syncope	1 (12.5%)	2 (25.0%)
History of atrial fibrillation	1 (12.5%)	2 (25.0%)
History of heart failure	2 (25.0%)	2 (25.0%)
Anti-arrhythmic drugs	2 (25.0%)	2 (25.0%)
Holter		
Time between Holter and CMR (m)	−0.3 (−2.0–0.8)	−2.2 (−8.0–8.5)
PVC burden (%)	0.9 (0.1–14.4)	0.7 (0.1–5.0)
nsVT burden (n/24 h)	0 (0–1)	2 (0–10)
Indication CMR		
Left ventricular remodeling	5 (62.5%)	3 (37.5%)
Arrhythmia	3 (37.5%)	5 (62.5%)
MVP	7 (87.5%)	7 (87.5%)
Posterior only	2 (25.0%)	2 (25.0%)
Both leaflets	5 (62.5%)	5 (62.5%)
Anterior MVP in mm	4.1 (3.5–5.0)	4.0 (3.0–7.4)
Posterior MVP in mm	4.9 (4.0–7.5)	6.6 (5.5–8.0)
MAD	8 (100%)	8 (100%)
MAD length (mm)	6.7 (5.0–9.0)	7.0 (6.0–12.0)
LA area (cm^2^/m^2^)	13.3 (10.1–15.6)	13.5 (11.3–17.3)
LVEF (%)	48.5 (32.0–57.0)	52.5 (44.0–59.0)
LV GLS	17.0 (9.7–21.0)	18.0 (13.1–20.4)
LV ESV (mL/m^2^)	58.0 (43.0–111.6)	56.2 (45.8–78.9)
LV curling (%)	7 (87.5%)	8 (87.5%)
Mitral regurgitation		
Grade 0	1 (12.5%)	2 (25.0%)
Grade 1	5 (62.5%)	3 (37.5%)
Grade 2	1 (12.5%)	3 (37.5%)
Grade 3	1 (12.5%)	0 (0.0%)
Late gadolinium enhancement		
Any LGE	3 (37.5%)	5 (62.5%)
% LGE	0.3 (0.3–1.5)	1.6 (0.7–2.4)
Inferolateral myocardium	1 (12.5%)	3 (37.5%)
Papillary muscle	2 (25.0%)	4 (50.0%)
Time since baseline CMR (y)	4.7 (2.8–7.7)	
New late gadolinium enhancement	4 (50.0%)	
Inferolateral myocardium	2 (50.0%)	
Papillary muscles	3 (75.0%)	
Clinical evolution		
No arrhythmias	1 (12.5%)	
Catheter ablation for PVC ectopy	1 (12.5%)	
Increased burden/presyncope/syncope	4 (50.0%)	
Negative PVS—suppressed with BB	2 (50.0%)	
Positive PVS—ICD implanted	2 (50.0%)	

Continuous variables are presented as median with range (minimum–maximum). Abbreviations: BB: beta-blocker; ICD: implantable cardioverter defibrillator; LGE: late gadolinium enhancement; LVEF: left ventricular ejection fraction; m: months; MAD: mitral annular disjunction; mm: millimeter; MVP: mitral valve prolapse; nsVT: non-sustained ventricular tachycardia; PVC: premature ventricular complexes; PVS: programmed ventricular stimulation; and y: year.

**Table 2:** Changes in CMR and clinical evolution in patients with mitral valve surgery.

Variable	Baseline CMR	Follow-Up CMR
Age (y)	38.6 (36–55.1)	41.6 (37.0–56.8)
Female	3 (75.0%)	
History of syncope	0 (0.0%)	0 (0.0%)
History of atrial fibrillation	0 (0.0%)	0 (0.0%)
History of heart failure	2 (50.0%)	2 (50.0%)
Anti-arrhythmic drugs	0 (0.0%)	0 (0.0%)
Holter		
Time between Holter and CMR (m)	−1.3 (−2.9–0.1)	0.7 (−5.7–2.9)
PVC burden (%)	4.8 (0.5–6.3)	4.1 (24.3–0.2)
nsVT burden (n/24 h)	2 (0–3	2 (0–569)
Indication CMR		
Left ventricular remodeling	0 (0.0%)	2 (50.0%)
Arrhythmia	4 (100%)	2 (50.0%)
MVP	4 (100%)	0 (0.0%)
Posterior only	1 (25.0%)	
Both leaflets	3 (75.0%)	
Anterior MVP in mm	4.0 (3.0–6.0)	
Posterior MVP in mm	7.5 (6.0–9.0)	
MAD	2 (50.0%)	0 (0.0%)
MAD length (mm)	7.0 (5.0–9.0)	
LA area (cm^2^/m^2^)	15.9 (10.8–21.7)	12.6 (9.1–22.2)
LVEF (%)	50.0 (39.0–59.0)	48.5 (43.0–50.0)
LV GLS	16.1 (10.0–18.2)	15.3 (14.3–16.4)
LV ESV (mL/m^2^)	68.8 (57.1–78.7)	55.3 (48.4–70.8)
LV curling (%)	4 (100%)	0 (0.0%)
Mitral regurgitation		
Grade 0	0 (0.0%)	0 (0.0%)
Grade 1	0 (0.0%)	4 (100%)
Grade 2	0 (0.0%)	0 (0.0%)
Grade 3	4 (100%)	0 (0.0%)
Late gadolinium enhancement		
Any LGE	1 (25.0%)	2 (50.0%)
% LGE	0.6	0.9 (0.5–2.2)
Inferolateral myocardium	1 (25.0%)	2 (50.0%)
Papillary muscle	0 (0.0%)	0 (0.0%)
Time since baseline CMR (y)	1.5 (0.8–4.5)	
New late gadolinium enhancement	2 (50.0%)	
Inferolateral myocardium	2 (100%)	
Papillary muscles	0 (0.0%)	
Clinical evolution		
No arrhythmias	1 (25.0%)	
PVC burden decrease on BB	1 (25.0%)	
Increased PVC burden despite BB	2 (50.0%)	

Continuous variables are presented as median with range (minimum–maximum). Abbreviations: BB: beta-blocker; LGE: late gadolinium enhancement; LVEF: left ventricular ejection fraction; m: months; MAD: mitral annular disjunction; mm: millimeter; MVP: mitral valve prolapse; PVC: premature ventricular complexes; and y: year.

## Data Availability

The raw data supporting the conclusions of this article will be made available by the authors on request.

## Supplementary Materials

The following supporting information can be downloaded at: https://www.mdpi.com/article/10.3390/jcm13092669/s1. Figure S1. Bland-Altman analysis for intra- and interobserver variability of LGE measurement. Table S1. Comparison of patients with 1 CMR and those with >1 CMR included in this analysis. Figure S2. Example 2 of new LGE on serial CMR in a patient without surgical intervention. Figure S3. Change in LGE between baseline and follow-up CMR. Figure S4. Example 2 of new LGE on serial CMR in a patient with surgical intervention.
