# Supplementary material for: Serial Cardiac Magnetic Resonance Imaging in Patients with Mitral Valve Prolapse—A Single-Center Retrospective Registry
Source: J Clin Med. 2024 May 2;13(9):2669. doi: 10.3390/jcm13092669 (PMC11084857; doi:10.3390/jcm13092669)

Figure S1. Bland-Altman analysis for intra- and interobserver variability of LGE measurement.

A. Intra-observer variability

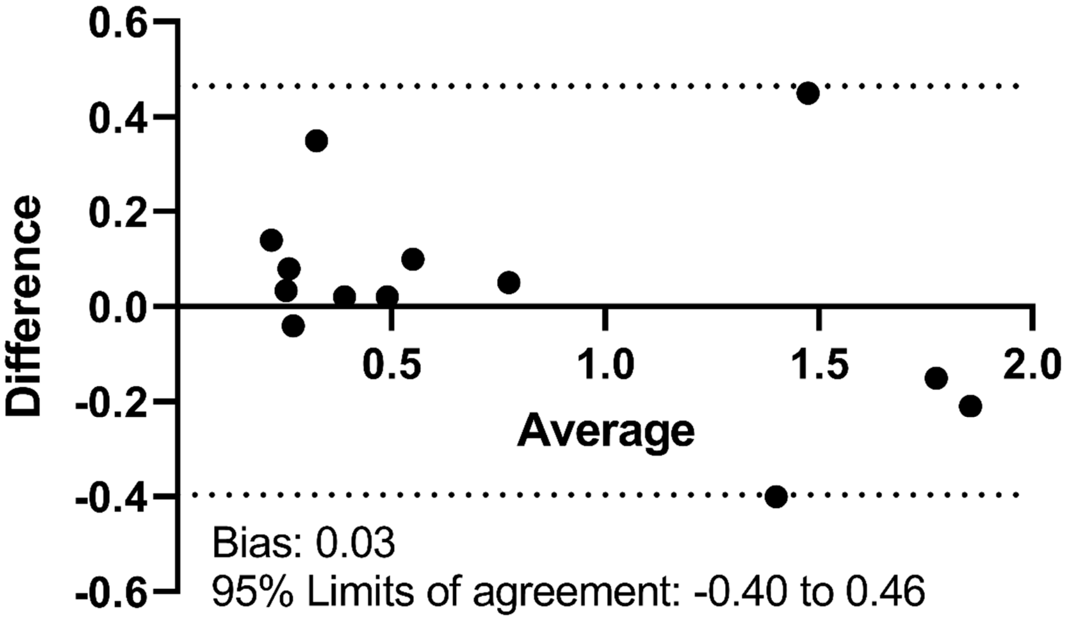

B. Interobserver variability

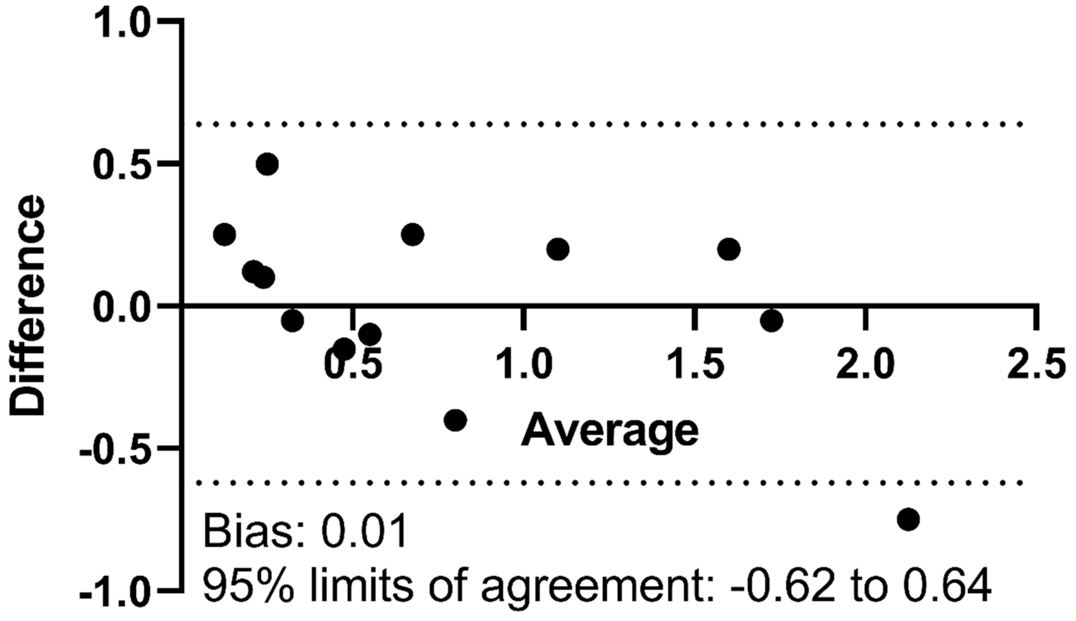

Table S1. Comparison of patients with 1 CMR and those with >1 CMR included in this analysis.

Variable	All patients (N=169)	Single CMR (N=157)	Serial CMR (N=12)	p-value
Age – first cardiac contact (y)	44.3 (29.8 – 56.9)	46.0 (30.8 – 58.1)	34.0 (20.2 – 45.7)	0.052
Age – CMR (y)	51.4 (36.5 – 62.4)	51.5 (35.7 – 63.0)	43.8 (33.3 – 56.8)	0.177
Follow-up since first cardiac contact (y)	7.9 (3.8 – 15.7)	7.8 (3.7 – 15.2)	14.4 (8.2 – 20.8)	0.019
Male	88 (52.1%)	85 (54.1%)	3 (25.0%)	0.072
BMI (kg/m²)	22.8 (20.6 – 24.9)	22.8 (20.6 – 24.9)	23.5 (20.2 – 25.2)	0.770
Arterial hypertension	27 (16.0%)	25 (15.9%)	2 (16.7%)	1.000
Dyslipidemia	52 (30.8%)	50 (31.8%)	2 (16.7%)	0.347
Active smoking	35 (20.7%)	34 (21.7%)	1 (8.3%)	0.463
Heart failure	13 (7.7%)	10 (6.4%)	3 (27.3%)	0.042
Marfan	18 (10.7%)	17 (10.8%)	1 (8.3%)	1.000
Familial SCD	22 (13.0%)	19 (12.1%)	3 (25.0%)	0.193
Familial MVP	15 (8.9%)	13 (8.3%)	2 (16.7%)	0.289
Palpitations	115 (68.0%)	106 (67.5%)	9 (75.0%)	0.754
Syncope	23 (13.6%)	22 (14.0%)	1 (8.3%)	1.000
SCD	4 (2.4%)	4 (2.5%)	0 (0.0%)	1.000
Beta-blocker	85 (50.6%)	76 (48.4%)	9 (81.8%)	0.057
Non-dihydropyridine CCB	7 (4.2%)	7 (4.5%)	0 (0.0%)	1.000
Anti-arrhythmic drugs				
Sotalol	4 (2.4%)	3 (1.9%)	1 (8.3%)	0.257
Class Ic	10 (5.9%)	9 (5.7%)	1 (8.3%)	0.531
Amiodarone	2 (1.2%)	2 (1.3%)	0 (0.0%)	1.000
ACE/ARB/ARNI	32 (19.0%)	28 (17.8%)	4 (36.4%)	0.224
Barlow's disease	56 (33.3%)	51 (32.5%)	5 (45.4%)	0.509
Heart rate (bpm)	65.0 (57.0 – 78.0)	65.0 (58.0 – 78.0)	65.0 (55.3 – 73.3)	0.481
PR interval (ms)	158.0 (140.0 – 178.0)	158.0 (141.0 – 178.0)	145.0 (136.5 – 182.0)	0.447
QRS duration (ms)	94.0 (86.0 – 103.0)	94.0 (86.0 – 104.0)	97.0 (88.0 – 102.0)	0.486
QTc duration (ms)	413.0 (403.0 – 426.0)	413.0 (402.0 – 426.0)	421.0 (403.3 – 433.0)	0.419
PVC on any ECG	68 (41.5%)	59 (38.8%)	9 (75.0%)	0.029
nsVT on any ECG	3 (1.8%)	3 (2.0%)	0 (0.0%)	1.000
Fragmented QRS	24 (14.6%)	23 (15.1%)	1 (8.3%)	1.000
Inferior TWI	61 (36.1%)	54 (34.4%)	7 (58.3%)	0.122

Continuous variables are presented as median with quartiles 1 and 3.

Abbreviations: ACE/ARB/ARNI: angiotensin-converting enzyme inhibitor / angiotensin receptor blocker / angiotensin receptor-neprilysin inhibitor; BMI: body mass index; bpm: beats per minute; CCB: calcium channel blocker; CMR: cardiac magnetic resonance imaging; ECG: electrocardiogram; ms: milliseconds; MVP: mitral valve prolapse; nsVT: non-sustained ventricular tachycardia; PVC: premature ventricular complex; SCD: sudden cardiac death; TWI: T-wave inversion; y: year.

Figure S2. Example 2 of new LGE on serial CMR in a patient without surgical intervention.

A. Short-axis view on the baseline CMR at the level of the PM shows LGE in the posterior pappillary muscle (red arrow).

B. Short-axis view at the same level approximately 6 years later shows LGE in the posterior pappillary muscle (red arrow) and new subendocardial LGE in the inferolateral left ventricular wall (blue arrow).

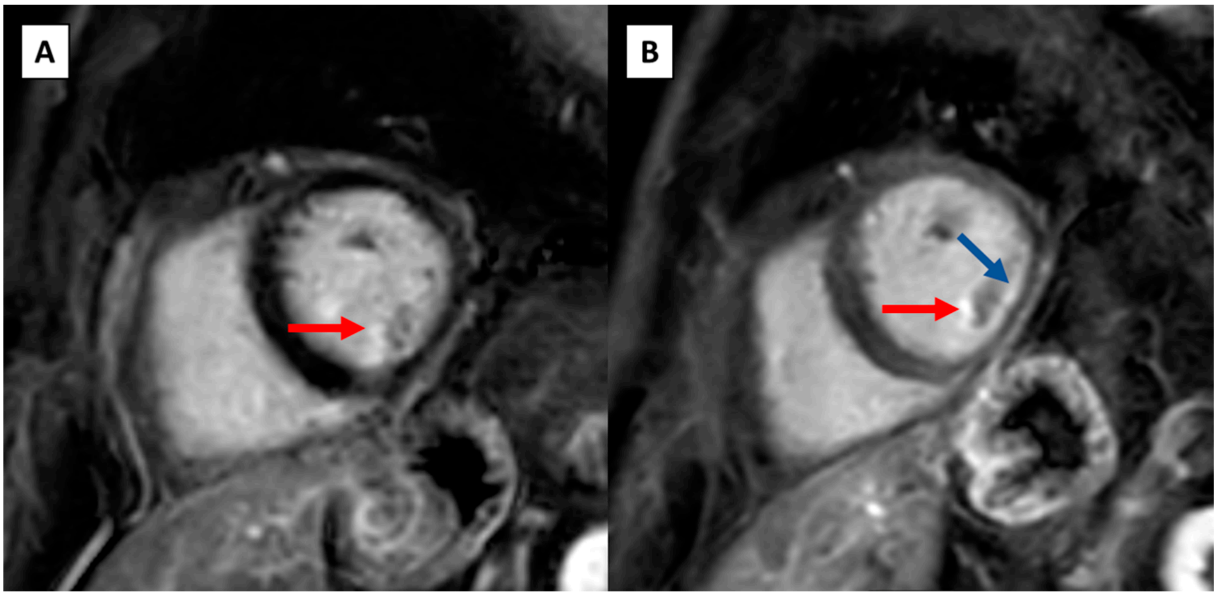

Figure S3. Change in LGE between baseline and follow-up CMR.

A. Myocardial LGE in patients without surgical intervention.

B. Myocardial LGE in patients with surgical intervention.

C. Papillary muscle LGE in patients without surgical intervention.

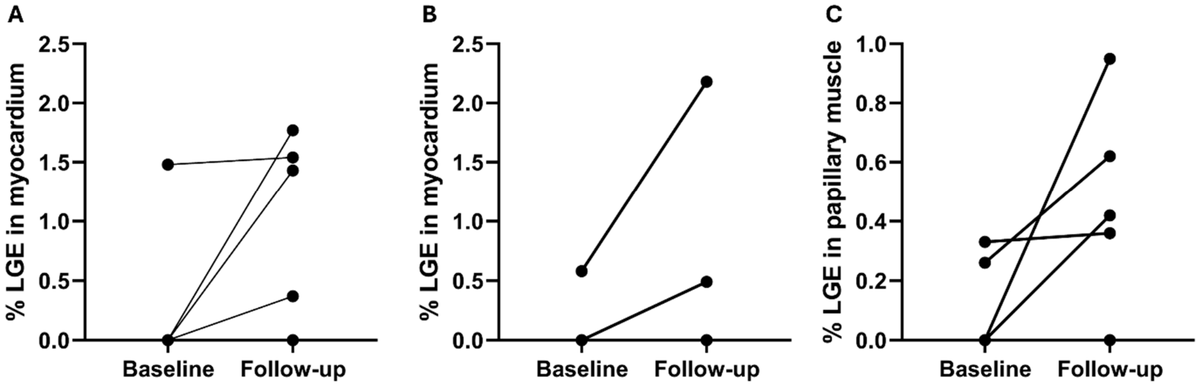

Figure S4. Example 2 of new LGE on serial CMR in a patient with surgical intervention.

A. Short-axis view on the baseline CMR.

B. Short-axis view at the same level shows new LGE in the basal inferolateral left ventricular wall (red arrow).

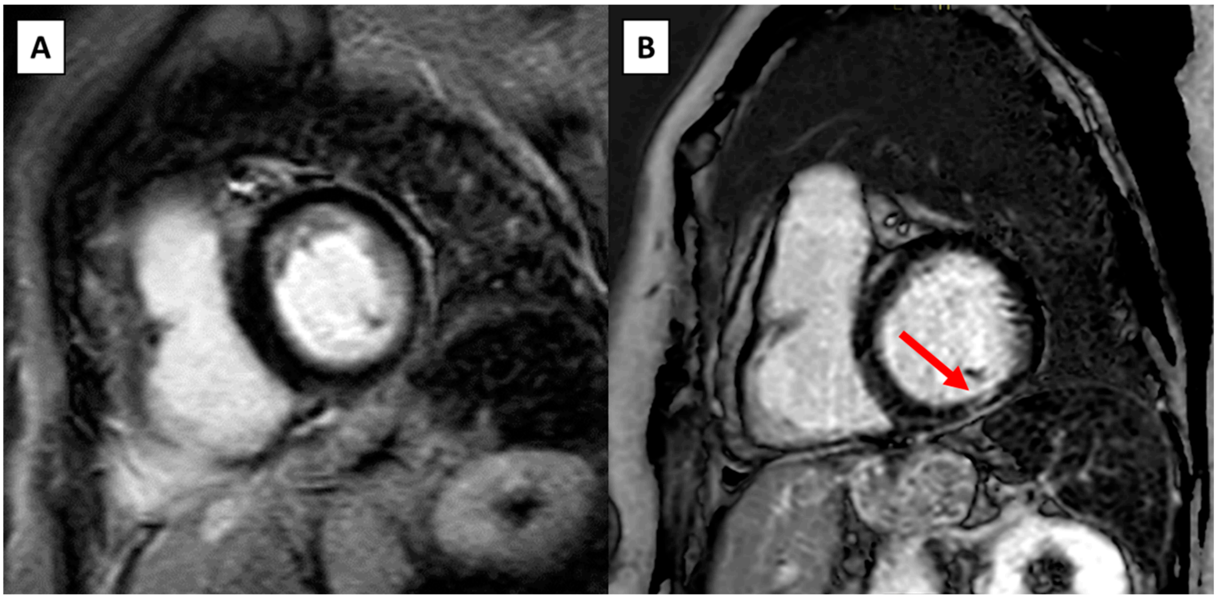

Supplement: Supplementary file 1 [file jcm-13-02669-s001.zip › jcm-2944763-supplementary.pdf]
